# Accurate isoform discovery with IsoQuant using long reads

**DOI:** 10.1038/s41587-022-01565-y

**Published:** 2023-01-02

**Authors:** Andrey D. Prjibelski, Alla Mikheenko, Anoushka Joglekar, Alexander Smetanin, Julien Jarroux, Alla L. Lapidus, Hagen U. Tilgner

**Affiliations:** 1 Center for Algorithmic Biotechnology, Institute of Translational Biomedicine, St. Petersburg State University, St. Petersburg, Russia; 2 Department of Computer Science, University of Helsinki, Helsinki, Finland; 3 Tri-Institutional Computational Biology and Medicine, Weill Cornell Medicine, New York, NY USA; 4 Brain and Mind Research Institute, Weill Cornell Medicine, New York, NY USA; 5 Center for Neurogenetics, Weill Cornell Medicine, New York, NY USA; 6 Bioinformatics Institute, St. Petersburg, Russia

**Keywords:** Genome informatics, Software

## Abstract

Annotating newly sequenced genomes and determining alternative isoforms from long-read RNA data are complex and incompletely solved problems. Here we present IsoQuant—a computational tool using intron graphs that accurately reconstructs transcripts both with and without reference genome annotation. For novel transcript discovery, IsoQuant reduces the false-positive rate fivefold and 2.5-fold for Oxford Nanopore reference-based or reference-free mode, respectively. IsoQuant also improves performance for Pacific Biosciences data.

## Abstract (web summary)

IsoQuant predicts novel isoforms from long-read RNA sequencing.

## Main

Long-read RNA sequencing is now widely used in bulk, sorted cells, single cells and spatial approaches. This wide field of applications has led to the development of multiple spliced alignment programs^1–4^, transcript discovery methods^5–11^, tools for transcript classification^12^, annotation^13^ and visualization^14,15^. Additionally, several reference-free tools for RNA long-read correction and assembly have been developed^16,17^. Current community efforts address the problem of understanding performance, weaknesses and advantages of each approach for various applications^18^.

Here we present IsoQuant—a tool for transcript discovery and quantification with long RNA reads. IsoQuant takes as input a reference genome and a dataset containing PacBio or ONT (Oxford Nanopore Technologies) RNA reads. By default, IsoQuant maps input reads to the genome via minimap2 in splice mode^2^. Alternatively, a user may provide BAM files generated with a spliced aligner of their choice, for example STARlong^1^ for PacBio and uLTRA^4^ or deSALT^3^ for ONT reads. In two distinct modes, IsoQuant can be used for de novo annotation-free transcript discovery as well as with the reference gene annotation.

**Fig. 1:**
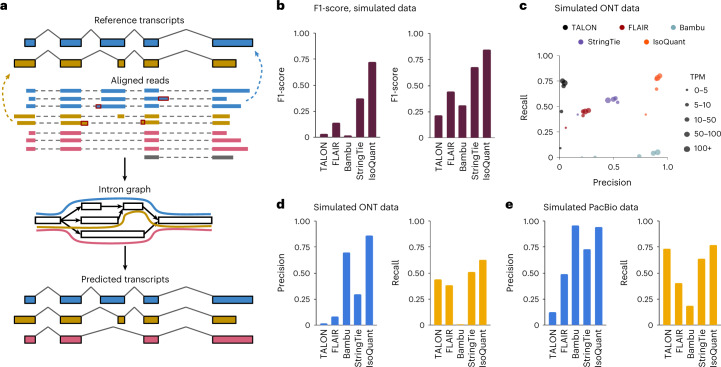
IsoQuant pipeline outline and characteristics of novel transcripts generated from mouse simulated data. **a**, Outline of the IsoQuant pipeline. When a reference gene annotation is provided, reads are assigned to annotated isoforms and alignment artifacts are corrected (top). The intron graph is constructed from read alignments (middle) and transcripts are discovered via path construction (bottom). **b**, F1-score for novel transcripts reported by different tools on simulated ONT (left) and PacBio data (right). **c**, Precision and recall for novel transcripts reported by different tools on simulated ONT data broken up by expression levels in TPM. TPM bins are presented by dot sizes. **d**, Precision (left) and recall (right) for novel transcripts reported by different tools on simulated ONT data. **e**, Same as **d**, but for simulated PacBio data. Source Data

IsoQuant uses long-read spliced alignments to construct an intron graph, in which vertices are splice junctions, that is, pairs of splice sites (donor and acceptor), and two vertices are connected with a directed edge if the corresponding splice junctions are consecutive in at least one read (Methods). This graph is exploited for constructing paths that correspond to full-length transcripts (Fig. 1a). If the reference annotation is provided, IsoQuant first assigns reads to known isoforms via an inexact intron-chain matching algorithm that accounts for splice site shifts, which are typical for alignment of error-prone reads^19^. These assignments are further used for reference transcript quantification and correction of inaccurately detected splice junctions and misalignments, such as skipped microexons.

To compare IsoQuant performance against existing transcript discovery tools, we first simulated mouse PacBio and ONT data using realistic gene expression profiles with IsoSeqSim (https://github.com/yunhaowang/IsoSeqSim) and Trans-NanoSim^20^ respectively. For more informative benchmarking, we simulated an ONT R9.4 dataset representing R9.4 chemistry and an ONT R10.4 dataset corresponding to a more accurate R10.4 chemistry (Methods).

To mimic real-life datasets containing unannotated transcripts, we arbitrarily removed 5,311 (15%) of 35,684 expressed isoforms (the ones contributing to at least one read during the simulation) from the GENCODE^21^ gene annotation. These 5,311 hidden transcripts were further used as a ground truth for novel transcript discovery. The reduced GENCODE annotation was used as an input for all tools. Each output annotation was then separated into a set of known and a set of novel transcripts, which were compared against the respective baselines using gffcompare^22^ (Methods).

For known transcripts, IsoQuant has the highest F1-score (the harmonic mean of precision and recall) compared to TALON^7^, FLAIR^8^, Bambu^11^ and StringTie^5^, but these advances are not dramatic (Supplementary Tables 1–3). However, IsoQuant produces novel transcripts with a 1.9-fold higher F1-score on ONT R10.4 data compared to the second-best tool, StringTie. In comparison to TALON, FLAIR and Bambu, the improvement in F1-score is even more noticeable (Fig. 1b, left). On PacBio data, IsoQuant again shows the best F1-score, but the difference from other tools is smaller than for ONT R10.4 data (Fig. 1b, right).

Compared to most tools, IsoQuant’s improvements in F1-score is primarily caused by its very high precision of novel transcripts. As compared to TALON, FLAIR and StringTie, IsoQuant shows a minimum of fivefold drop in false-positive rate on ONT R10.4 data, while still maintaining slight gains in recall (Fig. 1d). The situation is of a different nature for Bambu. IsoQuant has higher precision (86.3 versus 69.9%), but substantially higher recall: while Bambu only reconstructs 73 out of 5,311 novel isoforms (1% recall), IsoQuant reconstructs 3,848 (62.6%). On ONT R9.4 simulated data IsoQuant similarly shows a notably lower false-positive rate compared to other tools (Supplementary Table 2).

On PacBio simulated data, similar trends can be observed for novel transcripts, although with a less drastic difference in specificity. Bambu shows slightly higher precision (95.8%) compared to IsoQuant (94.4%), but again has the lowest recall (18.7% for Bambu versus 76.8% for IsoQuant). StringTie, TALON and FLAIR again predict transcripts with comparable recall, but have at least fivefold higher false-positive rate compared to IsoQuant (Fig. 1e, detailed analysis of the false-positive transcript is provided in Supplementary Note 8).

Further, we measured precision and recall for novel transcripts with different expression levels (Fig. 1c and Supplementary Fig. 1). While all tools tend to show lower recall and precision for lowly expressed transcripts, IsoQuant yields highly specific transcript models (≥80% precision) and maintains advances for novel transcript discovery regardless of the expression levels. Thus, IsoQuant is likely to be highly useful across many genes, including but not limited to low-expressed long-noncoding RNAs and marker genes of cell types.

Among the five listed methods, only StringTie and IsoQuant support annotation-free transcript discovery. Thus, we compared these two tools on the same simulated datasets used above without providing any annotation (Supplementary Table 4). On PacBio data both tools yield highly accurate transcript models. On ONT data StringTie shows higher recall, while IsoQuant generates transcripts with substantially lower false-positive rates (2.5-fold decrease for ONT R10.4 dataset and 3.7-fold for ONT R9.4). While overall quality of transcripts discovered in reference-based mode is, indeed, higher compared to annotation-free runs, the precision and recall of novel transcripts appears to be rather similar in both modes.

To complement our benchmarks on simulated data, we also sequenced Lexogen spike-in RNA variant (SIRV) synthetic molecules on the Oxford Nanopore MinION using ONT R10.4 flowcells (Methods). Along with the complete SIRV annotation, Lexogen provides an incomplete annotation, missing 26 out of the total 69 SIRV isoforms, which allows the evaluation of novel transcript discovery, similar to the one we performed for simulated data with the reduced GENCODE annotation.

**Fig. 2:**
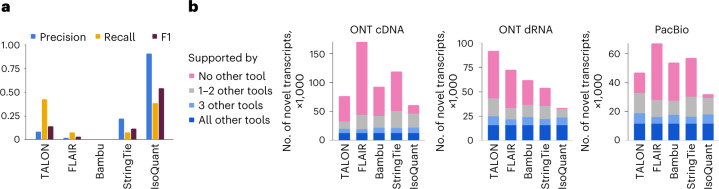
Characteristics of transcripts obtained from real sequencing data. **a**, Precision, recall and F1-score for novel transcripts generated on real SIRV ONT cDNA sequencing data. **b**, Consistency of predictions made by different methods on real human ONT cDNA, ONT dRNA and PacBio data. Source Data

Results on SIRV sequencing data resemble the ones obtained on simulated reads. When predicting novel isoforms, IsoQuant shows at least four times higher F1-score and eightfold lower false-positive rate than any other tool. In comparison to most tools, with the exception of TALON, IsoQuant shows high gains in both precision and recall. TALON has a better recall (42.3 versus 38.5%), but IsoQuant has tenfold higher precision (Fig. 2a). Similar to simulated data, all tools are able to accurately predict SIRV transcripts kept in the annotation, with Bambu, StringTie and IsoQuant having perfect precision for known isoforms alone (Supplementary Table 5).

To support our observations, we also applied all tools to the real human ONT complementary DNA, ONT direct RNA (dRNA)^23^ and PacBio public datasets, for which the ground truth is indeed unknown. We used gffcompare to estimate the consistency of predictions by computing the number of identical transcript models reported by the different tools. On the human ONT dRNA dataset, IsoQuant shows the highest percentage of transcripts confirmed by at least three other methods (70.1%), while no other tool surpasses the 40% threshold. This suggests that IsoQuant transcript models are notably more consistent with other methods (Fig. 2b, middle). In comparison to the other approaches, IsoQuant also reports the lowest number of transcripts that are not predicted by any other method. If one interprets such transcript models as potential false positives, IsoQuant again stands out in the lowest false-discovery rate (3.5%, 1,162 transcripts). In contrast, other tools output annotations containing more than 33% of unconfirmed transcript models (varying from 18,000 to 48,000). Additionally, for each tool we computed the number of potentially missed transcripts that were reported by all other methods. While TALON has the lowest number of such transcripts (75), Bambu shows the second-best results of 1,089 possible false negatives and IsoQuant shows the third-best results of 1,521 such transcripts (Supplementary Table 6).

Similar trends can be observed in ONT cDNA and PacBio datasets, although the overall percentage of common transcripts appears to be lower compared to ONT dRNA data (Fig. 2b, left and right). IsoQuant again shows the highest fraction of transcripts predicted by at least three other tools (35.6% for ONT cDNA, 55.6% for PacBio), while other programs have correspondingly 25 and 40% at best. All four other tools produce annotations containing a high number of transcripts that are not confirmed by any other method (> 50% of all transcripts for ONT cDNA, > 30% for PacBio), while IsoQuant’s potential false predictions are below 25% on ONT cDNA dataset and below 10% on the PacBio dataset.

Although these values cannot be explicitly treated as false positives and false negatives, they advocate that, unlike other tools, IsoQuant produces highly specific annotations that are strongly consistent with transcripts reported by several alternative approaches. Moreover, because IsoQuant typically misses very few isoforms predicted by all other tools simultaneously, it is likely to also be highly sensitive (Supplementary Table 6, the number of potentially missed transcripts).

Additionally, we used long-read RNA sequencing data from a mouse brain sample, in which a previous study reported 76 novel isoforms of high biological importance^24^, which were confirmed by manual annotation by the GENCODE team. Here, we compared IsoQuant only with StringTie, which has the second-best F1-score across all simulated datasets. On PacBio data, IsoQuant correctly reconstructs 71% of the confirmed novel isoforms, while StringTie restores approximately half as many novel transcripts—37% (Supplementary Table 7). Similarly, on the single-cell ONT dataset from the same brain sample IsoQuant restores almost 50% of these 76 novel isoforms, whereas StringTie reports 30%. Although it is not possible to evaluate specificity in this kind of experiment, it confirms that IsoQuant can maintain high recall values on real sequencing data.

Beside transcript discovery, IsoQuant implements additional functionality, such as read-to-isoform assignment and transcript quantification. Benchmarks of these supplementary features, information on computational performance, as well as IsoQuant results obtained with different spliced aligners can be found in the Supplementary Notes 2–7.

In summary, IsoQuant accurately predicts transcript models from PacBio or ONT RNA sequencing data. For known isoforms, IsoQuant has higher F1-score compared to other tested tools, but these differences are not dramatic. For unannotated isoforms, however, IsoQuant provides very strong increases in F1-score over other existing approaches. In comparison to most tools, it achieves this F1-score increase by maintaining higher recall, while substantially increasing precision. Thus, IsoQuant is a valuable tool for predicting novel alternatively spliced isoforms in the age of long-read sequencing.

## Methods

### Sequencing Lexogen SIRV transcripts

First, total RNA from HeLa cells was extracted using the miRNeasy Tissue/Cells Advanced Mini Kit (Qiagen, 217604), and polyA transcripts were pulled-down using the NEBNext Poly(A) messenger RNA Magnetic Isolation Module (NEB, E7490S). Next, the SIRV-Set 4 (Iso Mix E0/ERCC/Long SIRVs) (Lexogen, 141.01) was spiked-in to the RNA and reverse transcribed using the Maxima H Minus Reverse Transcriptase (Thermo Scientific, EP0752). The reverse transcriptase reaction final concentrations are as follows: 1.25 ng μl^−1^ polyA HeLa RNA, 0.33 ng μl^−1^ SIRV-Set 4, 0.5 mM dNTP, 5 μM dT-VN oligo, 5 μM TSO, 1× reverse transcriptase buffer, 2 U μl^−1^ RiboLock RNase Inhibitor (Thermo Scientific, EO0382) and 20 U μl^−1^ Maxima H Minus Reverse Transcriptase. The reaction was incubated for 30 min at 50 °C and 5 min at 85 °C. Then, 5 μl of reverse transcriptase reaction were amplified using the Platinum Superfi II Mastermix (ThermoFisher, 12368010) for 12 cycles, according to the manufacturer’s instructions and using Forward- and Reverse-Amplification primers. Finally, the cDNA was cleaned up using SPRIselect beads at a 0.8× ratio (Beckman Coulter, B23318) and used as input for Oxford Nanopore Technology sequencing with both the Kit 12 (SQK-LSK110 kit and FLO-MIN106D flowcells) and Q20+(SQK-LSK112 kit and FLO-MIN112 flowcells) chemistries. Both were run for 72 h and basecalled using the Super Accuracy model.

### Data simulation

To simulate PacBio circular consensus sequencing (CCS) reads we used IsoSeqSim (https://github.com/yunhaowang/IsoSeqSim), which generates a read by truncating a transcript sequence according to given probabilities and randomly inserts sequencing errors at a specified rate with uniform distribution. As reported in previous studies^25^, a uniform error distribution is a realistic model for PacBio CCS reads. Here we used 5′ and 3′ truncation probabilities typical for PacBio Sequel II (provided within the package) and an overall error rate of 1.6%: 0.6% deletions, 0.6% insertions and 0.4% substitutions. While these discrepancies do not necessarily represent sequencing errors, they must nevertheless be modeled, as they can confuse transcript reconstruction. The above values were obtained by mapping real PacBio CCS reads to the reference genome^18^.

ONT reads were simulated with the NanoSim software in the transcriptome mode^20^. NanoSim is designed specifically for simulating ONT-specific sequencing errors and biases. It first constructs error-profile and length-distribution models, which are further used to mutate reference transcript sequences. We trained the model using the ONT R10.4 sequencing data (average error rate of 2.8%: 0.7% deletions, 1.1% insertions, 1% substitutions.). To simulate ONT R9.4 chemistry, we used a pretrained model provided within the NanoSim package, which was obtained using publicly available ONT cDNA data^23^ from the NA12878 human cell line and has an average error rate of 15.9%: 6% deletions, 5.1% insertions and 4.8% substitutions. In addition, we turned off the simulation of intron retention events and random unaligned reads representing the background noise.

However, additional analysis of the simulated ONT data and NanoSim code revealed that NanoSim randomly selects a start position of a read in a transcript sequence with a uniform distribution, thus introducing no 5′ or 3′ bias. To simulate more realistic ONT reads, we aligned real ONT cDNA data obtained from the mouse brain sample to the reference transcriptome using minimap2 and derived empirical truncation probability distributions on both 5′ and 3′ ends. Further, we changed the NanoSim source code to enable sequence truncation with respect to obtained probabilities (Supplementary Fig. 2). The modified version is available at https://github.com/andrewprzh/lrgasp-simulation.

For both ONT and PacBio simulation we used Mouse GENCODE v.26 and Human GENCODE v.36 basic annotations^21^. Before simulation, we also attached a 30 basepair (bp) polyA tail to every transcript sequence. To simulate realistic mouse data, a transcript expression profile was obtained using PacBio data from a mouse brain sample^24^. For human data, a gene expression profile was computed with PacBio GM12878 data. A complete description of every dataset used in this study is provided in the Supplementary Table 8.

### Quality evaluation of predicted novel transcripts

To mimic real-life situations and assess the ability of an algorithm to predict novel transcripts, we created reduced gene annotations by removing a fraction of expressed isoforms. First, we define a subset of true expressed transcripts that contributed to at least one read during the simulation. Among this set, we select a fraction of transcripts to be excluded from the annotation. These transcripts are denoted as the true novel isoforms. The remaining transcripts (among the expressed) are defined as true known isoforms. To create a reduced gene annotation, we remove all true novel isoforms from the comprehensive GENCODE annotation. Here we created a reduced mouse annotation with 15% of expressed transcripts removed, and four human reduced annotations with 10, 15, 20 and 25% of excluded expressed isoforms (Supplementary Note 2).

To evaluate a transcript prediction tool, we provided the entire set of simulated reads and the reduced annotation as an input. Thus, true novel isoforms are hidden from the annotation, but present in the reads. We then compute precision and recall by running gffcompare^22^ for (1) the entire output annotation versus the complete set of expressed transcripts, (2) reported known isoforms versus the set of true known isoforms and (3) predicted novel transcript models versus the true novel set. The information on whether a transcript is known or novel is obtained from the output GTF file. The script for computing these metrics can be found in the IsoQuant repository in misc/reduced_db_gffcompare.py.

For the annotation-free benchmarks we simply compared the entire output annotation with the true set of expressed isoforms using gffcompare.

To estimate how recall and precision of novel transcripts depend on the expression levels, predicted transcripts are grouped into bins by their transcripts per million (TPM) values. For computing recall the number of false negative calls (undetected transcripts) in each TPM bin is required. We thus group transcripts by their TPM values used during the simulation. However, computing precision requires the number of false-positive predictions within each bin and thus only reported TPM values can be used (the true TPM for a false prediction is 0). Thus, it may happen that the same transcript may fall into different bins when benchmarking different tools. Although it is not possible to compute precision and recall exactly for an arbitrary TPM range, the bias has a minor effect as only a small number of bins was used in this experiment (five). Therefore, despite being imperfect, these estimations can provide additional insights on whether a transcript discovery method has any bias toward high- or low-expressed isoforms.

To evaluate SIRV transcripts we used an incomplete SIRV annotation containing only 43 out of 69 SIRV transcripts. The output annotations were again split into known and novel transcripts, and compared against the respective reference set using gffcompare. The SIRV-Set 4 annotations are available at https://www.lexogen.com/sirvs/download/.

### Estimating consistency between annotations

Consistency between transcripts generated on real data was estimated using gffcompare (without providing a reference annotation). Based on gffcompare output, for each tool we computed how many of its transcripts are supported by (1) all four other tools, (2) exactly three other tools, (3) one or two other tools and (4) no other tool (possible false predictions). We also counted the number of potentially missed transcripts that were reported by all methods except the one being evaluated (possible false negative). This approach is implemented in misc/denovo_model_stats.py.

### Command line options

For PacBio data minimap2 was launched with ‘splice:hq’ preset; for ONT data we used *k*-mer size 14 with the usual ‘splice’ preset. We also provided annotated splice junctions in BED format as an input. In each experiment, all tools were provided with the same BAM file and the same reference annotation. IsoQuant was launched with the default parameters setting the appropriate data type via ‘–data_type’ option. StringTie2 was launched with the ‘-L’ option. All other tools were run with the default parameters in 20 threads. In contrast to all other tools, Bambu outputs all reference transcripts, including unexpressed ones. Thus, we filtered out all transcripts with read count values <1 from the Bambu output. As recommended in the user manual, we also ran TALON using preliminary alignment correction with TranscriptClean^26^ (https://github.com/mortazavilab/TALON). However, as the results with and without correction were almost identical, we decided to use the annotations obtained from raw data for a fair comparison. Complete information on all options and software versions are provided in the Supplementary Table 9.

### IsoQuant algorithm

To process long RNA reads, IsoQuant requires a reference genome and optionally—a corresponding gene annotation. If the reads are provided in the FASTQ format, IsoQuant maps them to the reference with minimap2 in splice mode^2^. Alternatively, a user may provide a sorted and indexed BAM file generated with a spliced aligner of their choice. If the reference annotation is provided, the IsoQuant algorithm includes four main steps: (1) assigning mapped reads to known isoforms, (2) transcript quantification, (3) alignment correction and (4) transcript model construction. In the annotation-free mode, the pipeline simply proceeds to the transcript discovery step. Below, we describe the key aspects of all four procedures.

### Assigning long reads to known isoforms

The algorithm for assigning long reads to annotated isoforms is based on intron-chain matching and detecting exonic overlaps. To assign reads, IsoQuant processes each gene individually by extracting reads that map to the respective region from the sorted BAM file.

IsoQuant first processes the annotation to construct splice junction and exon profiles of all known isoforms. A set of annotated splice junctions in the gene is sorted according to their coordinates in the genome and enumerated from 1 to *N*. Thus, an annotated isoform can be represented as a vector of length *N*, in which the element at position *i* is set to 1 if this isoform includes the *i*th splice junction and −1 otherwise (Supplementary Fig. 3a). This vector is henceforth referred to as an isoform splice junction profile. The exon profile is constructed in a similar manner: all annotated exons are first split into a minimal set of *M* nonoverlapping fragments, such that every exon can be represented as their combination, and these exonic fragments are sorted and enumerated. The exon profile for an annotated isoform is similarly denoted as a vector of length *M*, where the *i*th element is set to 1 if this isoform contains the *i*th exon fragment and −1 otherwise (Supplementary Fig. 3b).

To assign a read to an annotated isoform, each splice junction from the alignment is matched against annotated splice junctions from the current gene and a read splice junction profile is constructed (also a vector of length *N*). In this vector the *i*th element is set to 1 if the annotated splice junction with index *i* matches to a splice junction from the read, −1 if it is overlapped or spanned by the read, but no match is detected, and 0 otherwise. A zero value indicates that the splice junction is located outside the alignment region and therefore no information can be derived, for example due to read truncation. Similarly, the exon profile of the read is constructed based on *M* exonic fragments described above: 1 indicates that the respective exonic fragment is overlapped, −1 means it is spanned and 0 is set for exonic fragments outside the alignment region (Supplementary Fig. 4).

Due to sequencing errors, an aligner may detect splice site positions inaccurately^19^. To avoid considering them as alternative or novel, the algorithm allows a small difference *Δ* between annotated and alignment splice site coordinates when matching splice junctions. Formally speaking, an annotated splice junction (*x*_1_, *x*_2_) matches a read splice junction (*y*_1_, *y*_2_) if |*x*_1_ − *y*_1_| ≤ *Δ* and |*x*_2_ − *y*_2_| ≤ *Δ*. The default *Δ* value varies for different types of input data: 4 bp used for PacBio CCS reads and 6 bp for ONT reads (can be set manually). Although an aligned read can be assigned to an isoform by simply comparing its intron chain and exonic coordinates to the annotation, vectorizing the alignment as described above allows one to easily implement inexact splice site comparison with a delta, and quickly detect candidate isoforms for read assignment.

Further, to assign a read to an isoform, its exon and splice junction profiles are matched against the respective profiles of the annotated isoforms. The distance between two profiles is computed simply as the number of distinct elements in which the read profile has nonzero values. A read is said to be consistent with an isoform if the distances between their exon and splice junction profiles are 0, and the read has no unannotated splice junctions/exons (Supplementary Fig. 4). When a read is consistent with a single isoform, it is reported as a unique match. When a read is consistent with multiple isoforms simultaneously, it is classified as ambiguous, which may happen, for example, due to read truncation. If a read contains unannotated splice junctions/exons, or its profiles are not consistent with any isoform, it is marked as inconsistent. For such alignments IsoQuant reports the most similar reference transcript and detected alternative splicing events.

Some inconsistencies can be, however, caused by misalignments, rather than by real alternative splicing events^19^: (1) skipped short exons, (2) intron shifts exceeding *Δ* bp and (3) short unannotated exons at transcript ends (Supplementary Fig. 5). If an inconsistent alignment contains only these types of discrepancy, the read is reclassified as conditionally consistent.

### Transcript quantification

Once long reads are assigned to annotated isoforms, quantification becomes rather trivial. Uniquely assigned reads are counted as a single detected transcript, while ambiguous reads are treated as multi-mappers and contribute to multiple assigned isoforms with lower weight. A transcript is reported as expressed only if it has at least one uniquely assigned read. Inconsistent reads are considered as potential novel isoforms and ignored during the quantification step. Beside genes and transcripts, IsoQuant can also count inclusion and exclusion abundances for separate exons and introns, which can be useful for computing percentage spliced-in values.

IsoQuant implements additional functionality for barcoded long RNA reads, for example barcoded by single-cell or spatial location^24,27^. A user can provide information on how the reads are grouped, for example, as a TSV file that indicates a barcode or a cell type of origin for every read. Isoform and gene abundances are then calculated for every read group separately, which can facilitate an expression comparison between different groups or cell types.

### Spliced alignment correction

IsoQuant corrects each uniquely assigned read individually. If a read contains misalignments described above (Supplementary Fig. 5) or its intron chain is not identical to the intron chain of the assigned isoform, the alignment is corrected as follows. Short skipped exons are restored according to the annotation and minor splice junction shifts are replaced with the respective splice junctions from the assigned transcript. Unannotated terminal microexons are simply removed from the alignment. Finally, any unannotated splice site is substituted with the nearest site from the assigned transcript if (1) these splice sites are located within *Δ* bp and (2) read alignment contains sequencing errors near this splice site. Coordinates of corrected alignments are then saved in BED12 format.

### Transcript model construction

The transcript reconstruction procedure implemented in IsoQuant includes four steps: (1) intron graph construction from read alignments, (2) intron graph simplification, (3) attaching terminal vertices and (4) construction of paths representing full-length transcripts. This stage does not require any information on reference transcripts and thus can be used for both de novo and annotation-based transcript discovery. Below we provide a detailed description of all algorithms and intuition behind them.

#### Intron graph construction

To construct transcript models, IsoQuant implements a concept of an intron graph, which was influenced by the previously designed splice graph approach^28^, used, for example, in StringTie^5^. For a given set of transcripts, an intron graph is constructed as follows. First, we define internal vertices as a set of all splice junctions from all transcripts. Thus, each vertex represents a pair of splice sites (donor and acceptor) or, more formally, an ordered pair of coordinates in the genome. Two vertices are connected with a directed edge if the respective splice junctions are consecutive in any transcript. Finally, for every first or last splice junction in a transcript, the corresponding vertex is connected with a terminating vertex that represents the transcript start and end positions (formally, a single integer). The intron graph is a directed acyclic graph since every edge connects only consecutive elements. Each transcript can now be represented as a path in the graph that traverses from the initial to terminal vertex, where internal vertices denote its intron chain (Supplementary Fig. 6a).

The described approach can be used to construct an intron graph from read alignments. Similarly, to the read-to-isoform assignment procedure, the genes are processed by IsoQuant individually. First, the algorithm constructs a set of internal vertices corresponding to splice junctions from the selected alignments. Two vertices are likewise connected when the respective splice junctions are consecutive in any read alignment. Due to the presence of inexactly detected splice sites, which may remain even after the alignment correction, such a graph may contain false vertices and connections. These false nodes typically form topological patterns, such as tips and bulges. A tip is defined as a dead end (dead start) edge that has a starting (ending) vertex with outdegree (indegree) at least 2. A bulge consists of two alternative paths having the same start and end vertices (Supplementary Fig. 6b). Similar patterns are also typical for de Bruijn graphs, which are used for short read assembly, where bulges and tips are caused by sequencing errors. To remove tips and bulges assemblers exploit various techniques broadly called graph simplification^29,30^.

#### Intron graph simplification

Here we implement a graph simplification procedure based on the following observations: (1) a false splice junction is typically unannotated, (2) splice site shifts that cause a false intron are short and (3) the number of reads supporting the correct splice junction often exceeds read support of a false one. Formally, a bulge/tip is removed from the graph if it represents an unannotated splice junction that has at least twice lower read support compared to the alternative vertex and the alternative vertex has splice sites within 20 bp (10 bp for PacBio). In other cases, when an unannotated splice junction has a high read support or no similar splice junction exists, a bulge or a tip is likely to represent a part of a novel isoform and thus should be preserved (Supplementary Fig. 6b). Although intron graph simplification strongly resembles naive splice junctions clustering, it has an important difference: a splice junction is removed not only based on its properties, such as splice site positions and read support, but based on the graph topology as well, thus considering adjacent splice junctions. Such a method allows one to, for example, preserve similar splice junctions from distinct isoforms. It is worth noting that the simplification procedure keeps track of all collapsed tips and bulges, thus preserving the possibility to later traverse alignment containing removed splice junctions through the graph.

#### Collecting terminal positions

After the graph is simplified, the algorithm proceeds to attach starting and terminal vertices. In contrast to annotated transcripts, read alignments do not provide the exact terminal positions, as their sequences can be truncated. Thus, to avoid having an extreme number of terminal vertices, terminal positions are detected using the heuristics presented below. Without loss of generality here we assume that the gene of interest is on the forward strand and polyA tails are on the right.

For every splice junction *V* in the graph, the algorithm selects only read alignments that contain *V* as a terminal splice junction and processes them as follows. First, the polyA sites are collected and clustered. Clustered polyA positions {*p*_1_, …, *p*_k_} are added to the graph as terminal vertices and connected to vertex *V* (Supplementary Fig. 7a). Further, the algorithm adds the rightmost non-polyA terminal position *P* as a terminal vertex if one of the conditions is satisfied: (1) *V* has no outgoing edges, (2) *V* has an outgoing edge to a splice junction (*u*_1_, *u*_2_) and *P* > *u*_1_ + *Δ* or (3) *V* has adjacent polyA vertices {*p*_1_, …, *p*_*k*_} and *P* > max(*p*_1_, …, *p*_*k*_) + *Δ* (where *Δ* is the parameter defined above). Thus, a non-polyA terminal position can only be attached if it is located to the right of adjacent exons or polyA vertices. Starting positions are collected in a similar manner, but without looking for polyA sites (Supplementary Fig. 7b). The described approach, however, may lose information when several isoforms share the same starting splice junction but have distinct transcription start and end sites. Thus, we also apply an additional transcripts correction, which is described below.

#### Transcript discovery via path construction

Once the intron graph is constructed and simplified, IsoQuant detects full-length paths that connect starting and terminal vertices. Paths entirely supported by at least a single read alignment (that is, full-splice match) are marked as transcript prediction candidates (Supplementary Fig. 7c). To filter out unreliable novel transcripts IsoQuant applies read support cutoffs: at least five full-splice match reads (three for PacBio) and at least 2% from the maximum graph coverage. Since some isoforms may not have a full-splice matching alignment, IsoQuant also reports known transcripts that (1) have at least one uniquely assigned read and (2) can be traversed through the intron graph. It also reports known mono-exonic transcripts that have (1) a uniquely assigned read and (2) a confirmed polyA site.

To correct terminal positions of a novel transcript, the algorithm selects all alignments consistent with this transcript and uses them to extract terminal positions using the approach described above (Supplementary Fig. 7d). In contrast to detecting terminal vertices for the entire graph, where all alignments are used, the subset of consistent reads likely belongs specifically to this isoform and thus provides correct start and end positions. The resulting transcripts are saved in GTF format, providing additional information about transcript types and their reference genes.

While the previously designed splice graph structure and the intron graph implemented in this work are designed to represent alternatively spliced transcripts and, in general, are highly similar, there are a few differences that can be highlighted. First of all, the splice graph natively supports transcription start and polyA sites as well as mono-exonic transcripts. The intron graph, however, requires the introduction of additional types of ‘terminal vertex’ that denote transcript start and end positions. At the same time, any exonic overlap between alternative transcripts will lead to a merged node in the splice graph, while the intron graph requires an exact match of both splice sites between two transcripts to form a single connected component. Thus, the intron graph can potentially be less tangled for the genes containing multiple alternatively spliced isoforms and, therefore, less complex to traverse through. Moreover, the intron graph natively provides information on neighboring splice junctions, which allows to easily detect incorrectly detected splice sites caused by misalignments and perform graph simplification. While this procedure can definitely be implemented within the splice graph concept, it seems to be more straightforward and native for the intron graph.

To evaluate how different steps of the transcript model construction algorithm affect recall and precision of IsoQuant, we performed a separate experiment described in Supplementary Note 1.

### Reporting summary

Further information on research design is available in the Nature Portfolio Reporting Summary linked to this article.

## Online content

Any methods, additional references, Nature Portfolio reporting summaries, source data, extended data, supplementary information, acknowledgements, peer review information; details of author contributions and competing interests; and statements of data and code availability are available at 10.1038/s41587-022-01565-y.

## Supplementary information

Supplementary Information Supplementary Tables 1–17, Figs 1–10 and Discussions.

Reporting Summary

## Data Availability

Nanopore sequencing data obtained from the human NA12878 cell line is available at https://github.com/nanopore-wgs-consortium/NA12878/blob/master/RNA.md. PacBio human GM12878 data is available at ENCODE (https://www.encodeproject.org/search) under the accession numbers ENCFF450VAU and ENCFF694DIE. Sequencing data obtained from mouse brain samples is available at NCBI Gene Expression Omnibus (https://www.ncbi.nlm.nih.gov/geo/) under accession numbers GSE158450 and GSE178175. ONT SIRV data, simulated data and reduced gene annotations are published at https://zenodo.org/record/7121404 (ref. ^31^).

## Source data

Source Data Fig. 1 Plots source data.
Source Data Fig. 2 Plots source data.

## Code availability

IsoQuant and the supplementary scripts used for the evaluation are available at https://github.com/ablab/IsoQuant. Scripts for data simulation are available at https://github.com/andrewprzh/lrgasp-simulation.
