# Supplementary material for: Accurate isoform discovery with IsoQuant using long reads
Source: Nat Biotechnol. 2023 Jan 2;41(7):915–8. doi: 10.1038/s41587-022-01565-y (PMC10344776; doi:10.1038/s41587-022-01565-y)
Supplement: Supplementary file 1 — Supplementary Tables 1–17, Figs 1–10 and Discussions. [file 41587_2022_1565_MOESM1_ESM.pdf]

nature biotechnology

Brief Communication https://doi.org/10.1038/s41587-022-01565-y

Accurate isoform discovery with IsoQuant using long reads

In the format provided by the authors and unedited

Supplementary Information

IsoQuant: a tool for accurate novel isoform discovery with long reads

Supplementary Tables 1

Supplementary Table 1. Results for ONT R10.4 mouse simulated data 1

Supplementary Table 2. Results for ONT R9.4 mouse simulated data 2

Supplementary Table 3. Results for PacBio mouse simulated data 3

Supplementary Table 4. Annotation-free transcript discovery on mouse simulated data 3

Supplementary Table 5. Results on real ONT data containing Lexogen SIRV Spike-ins 4

Supplementary Table 6. Consistency between annotations obtained from real data 5

Supplementary Table 7. Results on mouse brain sequencing data 6

Supplementary Table 8. Datasets used in this work 7

Supplementary Table 9. Command line options and software version used in this work 8

Supplementary Figures

Supplementary Figure 1. Novel transcripts obtained from ONT mouse simulated data 9

Supplementary Figure 2. Truncation probabilities for real and simulated ONT data 9

Supplementary Figure 3. Constructing isoform profiles 10

Supplementary Figure 4. Matching alignments against annotated isoforms 11

Supplementary Figure 5. Typical misalignments 11

Supplementary Figure 6. Intron graph construction 12

Supplementary Figure 7. Detection of terminal vertices and transcript reconstruction 13

Supplementary Discussions 14

Supplementary Note 1: Investigating IsoQuant algorithms 14

Supplementary Note 2: Varying fraction of hidden transcripts 16

Supplementary Note 3: Varying simulation parameters 17

Supplementary Note 4: Evaluating read-to-isoform assignment 19

Supplementary Note 5: Benchmarking transcript quantification 20

Supplementary Note 6: Testing IsoQuant with different spliced aligners 21

Supplementary Note 7: Computational performance 22

Supplementary Note 8: Analysis of incorrectly reconstructed novel isoforms 23

References 23

	Metric	TALON	FLAIR	Bambu	StringTie	IsoQuant
All	Total	164738	48470	41871	38216	31553
	Recall, %	59.0	70.6	80.0	81.1	**85**
	Precision, %	12.7	51.6	67.8	75.2	**95.6**
	F1-score	0.21	0.60	0.73	0.78	**0.90**
Known	Total	21298	24836	41798	29034	27705
	Recall, %	61.3	73.2	**93.4**	85.5	88.5
	Precision, %	87.1	89.2	67.6	89.1	**96.7**
	F1-score	0.72	0.80	0.78	0.87	**0.92**
Novel	Total	143440	23634	73	9182	3848
	Recall, %	44.0	38.5	1.0	51.2	**62.6**
	Precision, %	1.6	8.6	69.9	29.6	**86.3**
	F1-score	0.03	0.14	0.02	0.38	**0.73**

Supplementary Table 1. Results for ONT R10.4 mouse simulated data. All tools were launched using the same BAM file and the reduced gene annotation with 15% of expressed transcripts excluded. Results are provided for the following experiments. All transcripts: the entire output annotation is compared against the complete set of 35,684 expressed transcripts (both excluded and preserved in the reduced annotation); Known: compares output transcripts marked as known to a set of 30,373 expressed transcripts preserved in the annotation; Novel: compares transcript models marked as novel to a set of 5,311 transcripts hidden from the annotation. The best values are indicated with bold.

	Metric	TALON	FLAIR	Bambu	StringTie	IsoQuant
All	Total	178915	39515	43219	39524	32045
	Recall, %	64.5	69.4	80.6	82.1	**85.7**
	Precision, %	12.8	62.3	66.2	73.7	**94.9**
	F1-score	0.21	0.66	0.73	0.78	**0.90**
Known	Total	23967	22480	43110	30427	28134
	Recall, %	67.2	71.6	**94.1**	87	89.3
	Precision, %	84.8	**96.4**	66	86.5	96
	F1-score	0.75	0.82	0.78	0.87	**0.93**
Novel	Total	154948	17035	109	9097	3911
	Recall, %	47.1	37.9	1.3	49.6	**63.2**
	Precision, %	1.6	11.8	64.2	28.9	**85.7**
	F1-score	0.03	0.18	0.03	0.37	**0.73**

Supplementary Table 2. Results for ONT R9.4 mouse simulated data. All tools were launched using the same BAM file and the reduced gene annotation with 15% of expressed transcripts excluded. Results are provided for the following experiments. All transcripts: the entire output annotation is compared against the complete set of 35,684 expressed transcripts (both excluded and preserved in the reduced annotation); Known: compares output transcripts marked as known to a set of 30,373 expressed transcripts preserved in the annotation; Novel: compares transcript models marked as novel to a set of 5,311 transcripts hidden from the annotation. The best values are indicated with bold.

Transcripts	Metric	TALON	FLAIR	Bambu	StringTie	IsoQuant
All	Total	58777	26032	22180	31580	33549
	Recall, %	87.7	68.9	59.2	85.8	**93.4**
	Precision, %	53	93.9	94.6	96.3	**98.7**
	F1-score	0.66	0.79	0.73	0.91	**0.96**
Known	Total	27725	21634	21144	26948	29235
	Recall, %	89.9	71.2	65.9	88.4	**95.9**
	Precision, %	98	**99.5**	94.3	99.3	99.2
	F1-score	0.94	0.83	0.78	0.94	**0.98**
Novel	Total	31052	4398	1036	4632	4314
	Recall, %	73.4	40.5	18.7	63.6	**76.8**
	Precision, %	12.5	48.9	**95.8**	72.7	94.4
	F1-score	0.21	0.44	0.31	0.68	**0.85**

Supplementary Table 3. Results for PacBio mouse simulated data. All tools were launched using the same BAM file and the reduced gene annotation with 15% of expressed transcripts excluded. Results are provided for the following experiments. All transcripts: the entire output annotation is compared against the complete set of 35,684 expressed transcripts (both excluded and preserved in the reduced annotation); Known: compares output transcripts marked as known to a set of 30,373 expressed transcripts preserved in the annotation; Novel: compares transcript models marked as novel to a set of 5,311 transcripts hidden from the annotation. The best values are indicated with bold.

	PacBio		ONT R10.4		ONT R9.4	
	StringTie	IsoQuant	StringTie	IsoQuant	StringTie	IsoQuant
Transcripts	30,318	29,103	36,275	24,287	41,791	23,397
Recall, %	**80.1**	79.3	**65.1**	58.7	**62.2**	57.5
Precision, %	93.8	**96.7**	63.6	**85.7**	52.8	**87.3**
F1-score	0.86	**0.87**	0.64	**0.70**	0.57	**0.69**

Supplementary Table 4. Annotation-free transcript discovery on mouse simulated data. Comparison between StringTie and IsoQuant on 3 mouse simulated datasets: PacBio, ONT R10.4 and ONT R9.4. The true set used during the simulation contains 35,684 transcripts. The best values are indicated with bold.

	Metric	TALON	FLAIR	Bambu	StringTie	IsoQuant
All	Total	174	141	42	48	53
	Recall, %	63.8	47.8	60.9	59.4	**75.4**
	Precision, %	25.3	23.4	**100**	85.4	98.1
	F1-score	0.36	0.31	0.76	0.70	**0.85**
Known	Total	41	28	42	39	42
	Recall, %	76.7	60.5	**97.7**	90.7	**97.7**
	Precision, %	80.5	92.9	**100**	**100**	**100**
	F1-score	0.79	0.73	0.99	0.95	**0.99**
Novel	Total	133	113	0	9	11
	Recall, %	**42.3**	7.7	0	7.7	38.5
	Precision, %	8.3	1.8	0	22.2	**90.9**
	F1-score	0.14	0.03	0.00	0.11	**0.54**

Supplementary Table 5. Results on real ONT R10.4 data containing Lexogen SIRV Spike-ins. Results were obtained using ONT cDNA sequencing data and the incomplete SIRV annotation provided by Lexogen that has 26 out of 69 transcripts hidden. Results are provided for the following experiments. All transcripts: the entire output annotation is compared against the complete set of 69 SIRV transcripts; Known: compares output transcripts marked as known to a set of 43 SIRV transcripts preserved in the annotation; Novel: compares transcript models marked as novel to a set of 23 SIRV transcripts hidden from the annotation. The best values are indicated with bold.

Data	Transcripts	TALON	FLAIR	Bambu	StringTie	IsoQuant
ONT cDNA	# Total transcripts	76525	170670	92869	118803	61279
	Supported by all other tools, %	16.6	7.4	13.6	10.7	**20.7**
	Supported by 3 other tools, %	9.0	3.9	10.1	7.0	**14.9**
	Supported by 1-2 other tools, %	17.1	14.6	21.4	25.0	**39.5**
	Supported by no other tool, %	57.3	74.1	54.9	57.3	**24.9**
	# Potentially missed	3193	3408	**703**	1802	999
ONT dRNA	# Total transcripts	92023	72389	61971	53890	33310
	Supported by all other tools, %	17.0	21.6	25.2	29.0	**46.9**
	Supported by 3 other tools, %	10.0	7.9	13.2	11.5	**23.2**
	Supported by 1-2 other tools, %	20.1	16.6	20.5	25.1	**26.3**
	Supported by no other tool, %	52.9	53.9	41.1	34.3	**3.5**
	# Potentially missed	**75**	3533	1089	3040	1521
PacBio CCS	# Total transcripts	46914	67262	53931	57096	32088
	Supported by all other tools, %	24.5	17.1	21.3	20.2	**35.9**
	Supported by 3 other tools, %	15.3	6.8	11.6	8.4	**19.7**
	Supported by 1-2 other tools, %	30.0	17.8	18.2	24.3	**35.9**
	Supported by no other tool, %	30.2	58.3	48.9	47.1	**8.5**
	# Potentially missed	**97**	2718	1051	2464	951

Supplementary Table 6. Consistency between annotations obtained from real data. Percentages are given with respect to the total number of transcripts reported by the tool. The best values are indicated with bold.

Data	Metric	StingTie	IsoQuant
PacBio	Total transcripts	30554	33058
	Correct novel transcripts	28	**54**
	% of novel transcripts	36.8	**71.1%**
ONT spatial	Total transcripts	132441	72509
	Correct novel transcripts	20	**27**
	% of novel transcripts	26.3	**35.5%**
ONT single-cell	Total transcripts	186760	86187
	Correct novel transcripts	23	**37**
	% of novel transcripts	30.3	**48.7%**

Supplementary Table 7. Results on mouse brain sequencing data. Results of StringTie and IsoQuant on real PacBio and ONT mouse data. All datasets were initially sequenced as single-cell or spatial data, but treated as bulk in this experiment. The percentage of correct novel transcripts reported were computed against the set of 76 novel transcripts verified by the GENCODE team. As in the original study, a GENCODE v21 mouse comprehensive annotation was used in this experiment. The best values are indicated with bold.

<table>
<tr><th>Sample</th><th>Platform</th><th># reads, M</th><th>Properties</th><th>Accession number / link</th></tr>
<tr><td colspan="5">Simulated data</td></tr>
<tr><td rowspan="3">M. musculus</td><td>PacBio</td><td>6.0</td><td rowspan="5">Realistic expression profile</td><td rowspan="5">10.5281/zenodo.7121404</td></tr>
<tr><td>ONT cDNA R9.4</td><td>30.0</td></tr>
<tr><td>ONT cDNA R10.4</td><td>30.0</td></tr>
<tr><td rowspan="2">H. sapiens</td><td>PacBio</td><td>4.0</td></tr>
<tr><td>ONT cDNA</td><td>20.0</td></tr>
<tr><td colspan="5">Real data</td></tr>
<tr><td>Lexogen SIRVs</td><td>ONT cDNA</td><td>1.2</td><td></td><td>https://data.cab.spbu.ru/index.php/s/dgcaSaGME2xF7ed?path=%2FIsoQuant</td></tr>
<tr><td>H. sapiens GM12878</td><td>PacBio</td><td>4.7</td><td></td><td>ENCFF450VAU, ENCFF694DIE</td></tr>
<tr><td rowspan="2">H. sapiens NA12878</td><td>ONT cDNA</td><td>44.0</td><td></td><td rowspan="2">https://github.com/nanopore-wgs-consortium/NA12878/blob/master/RNA.md</td></tr>
<tr><td>ONT dRNA</td><td>25.2</td><td></td></tr>
<tr><td rowspan="3">M. musculus brain sample</td><td>PacBio</td><td>8.3</td><td rowspan="2">Single-cell data treated as bulk</td><td rowspan="2">GSE158450</td></tr>
<tr><td>ONT cDNA</td><td>31.4</td></tr>
<tr><td>ONT cDNA</td><td>144.9</td><td>Spatial data treated as bulk</td><td>GSE178175</td></tr>
</table>

Supplementary Table 8. Datasets used in this work.

Software	Version	Common options	PacBio options	ONT options
minimap2	2.18	-a -Y --junc-bed <junctions.bed> -t 20	-k 15 -x splice:hq	-k 14 -x splice
uLTRA	0.0.4.1	<annotation.gtf>	Not used	--ont
deSALT	1.5.6	aln -t 20	Not used	-l 14 -s 2 -x ont2d
TALON	5.0	-t 20		
FLAIR	1.5	-t 20		
Bambu	2.0.0	ncore=20		
StringTie	2.2.0	-L -p 20		
IsoQuant	3.0	--complete_genedb -t 20	-d pacbio_ccs	-d nanopore
SQANTI	4.2	sqanti3_qc.py --force_id_ignore --aligner_choice minimap2 --isoAnnotLite -t 20		
gffcompare	0.12.2			

Supplementary Table 9. Command line options and software versions used in this work. TALON, FLAIR, Bambu, StringTie, and IsoQuant were run using the same reference genome, reference annotation, and BAM file as an input. For the annotation-free benchmarks the same BAM files were used. Complete command lines are available in the IsoQuant repository in misc/all_comands.sh.

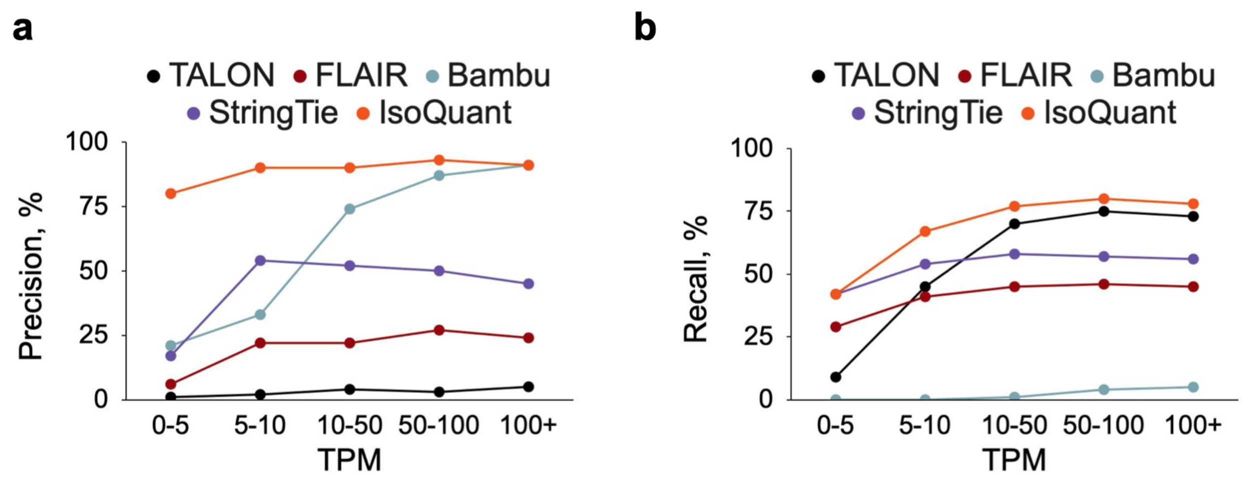

Supplementary Figure 1. Novel transcripts obtained from ONT mouse simulated data. a. Precision of novel transcripts generated by 5 tools with the respect to their TPM values. **b.** Recall of novel transcripts generated by 5 tools with the respect to their TPM values.

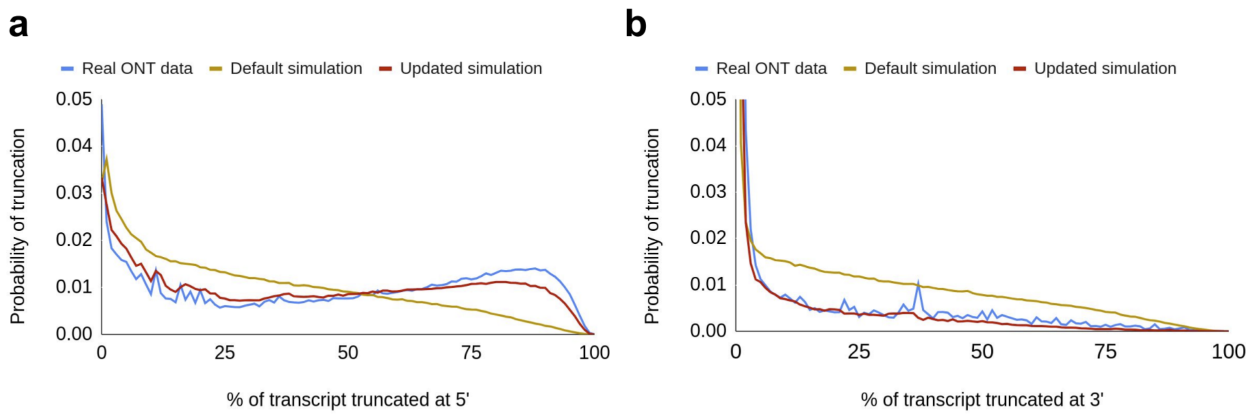

Supplementary Figure 2. Truncation probabilities for real and simulated ONT data. a. Empirical 5' truncation probability distributions for real ONT data from mouse brain sample (blue), data generated with the unmodified NanoSim (yellow), and data simulated using NanoSim with improved truncation procedure (red). The distributions were estimated by mapping reads onto the mouse reference transcriptome using minimap2 with -x map-ont option. **b.** Same as (a) but for the 3' end.

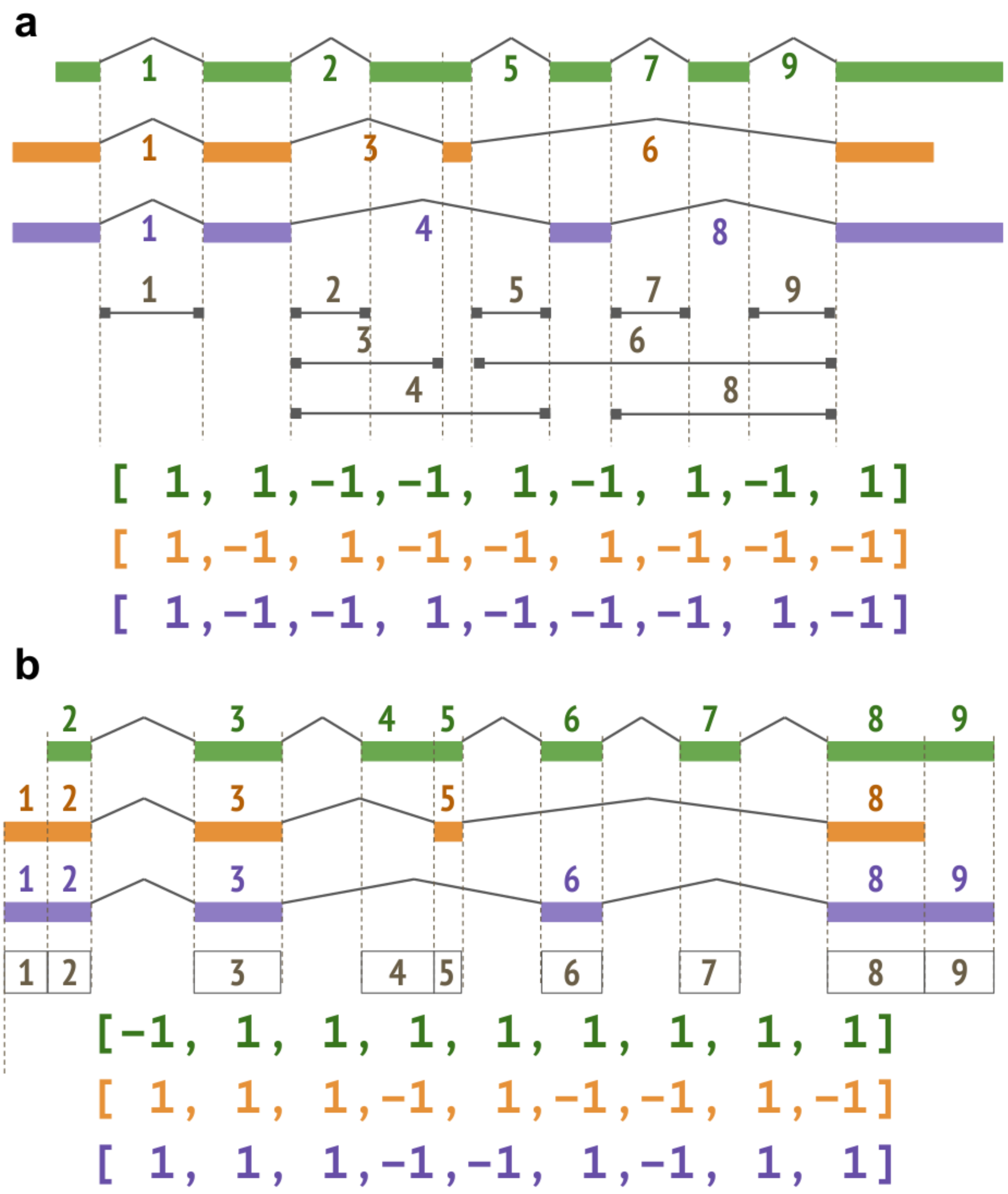

Supplementary Figure 3. Constructing isoform profiles. a. An example of splice junction profile construction for a gene having 3 isoforms (green, orange and purple). First, 9 annotated splice junctions are extracted and sorted by their coordinate (gray intervals in the middle). Each isoform is then represented as a vector of length 9 where each position specifies whether the respective splice junction is included in the isoform (1) or not (-1). **b.** An example of exon profile construction for the same gene as in (a). First, annotated exons are splitted into a minimal set of 9 non-overlapping fragments and sorted by their coordinate (gray bars in the middle). Each isoform is then represented as a vector of length 9 where each position specifies whether the respective part of the exon is covered by the isoform (1) or not (-1).

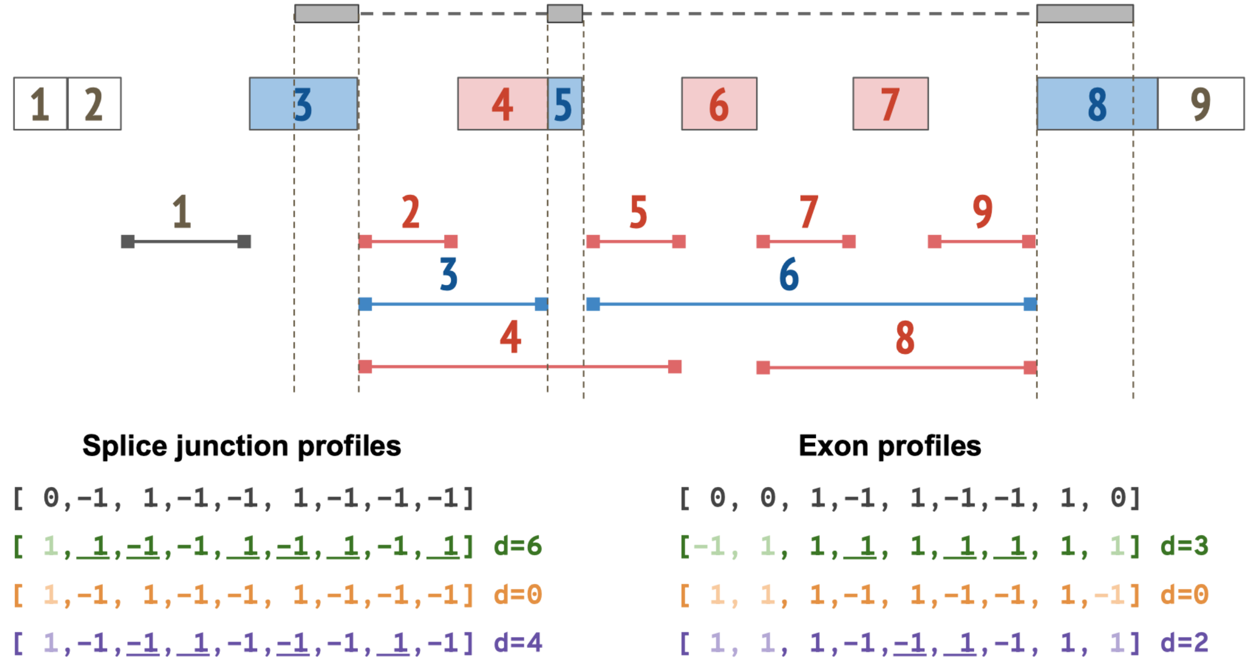

Supplementary Figure 4. Matching alignments against annotated isoforms. Exons and splice junctions from a read alignment (gray on top) are matched against the annotated exons and splice junctions. Known exons and splice junctions found in the alignment are colored blue, absent are highlighted with red. Read splice junction and exon profiles (gray vectors) are compared against the respective isoform profiles (colored vectors below) and distance between them is calculated as the number of distinct positions in which the read profile has a non-zero value.

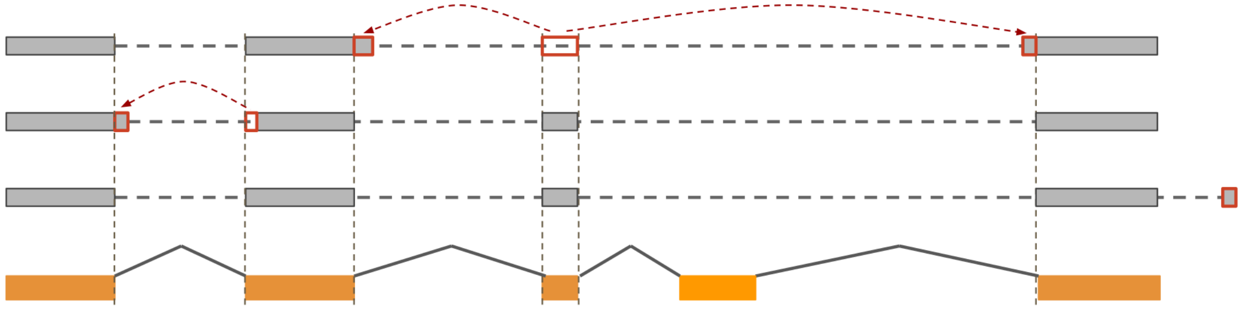

Supplementary Figure 5. Typical misalignments. Read alignments are colored gray, the correct original isoform is colored orange, misalignments are highlighted with red. A short exon is skipped by the alignment and its corresponding sequences are attached to the adjacent exons (top read). A 5’ fragment of the exon is incorrectly aligned near the 3’ end of the preceding exon, and thus the splice junction appears to be shifted (middle read). The alignment contains a false terminal microexon at 3’ end (bottom read).

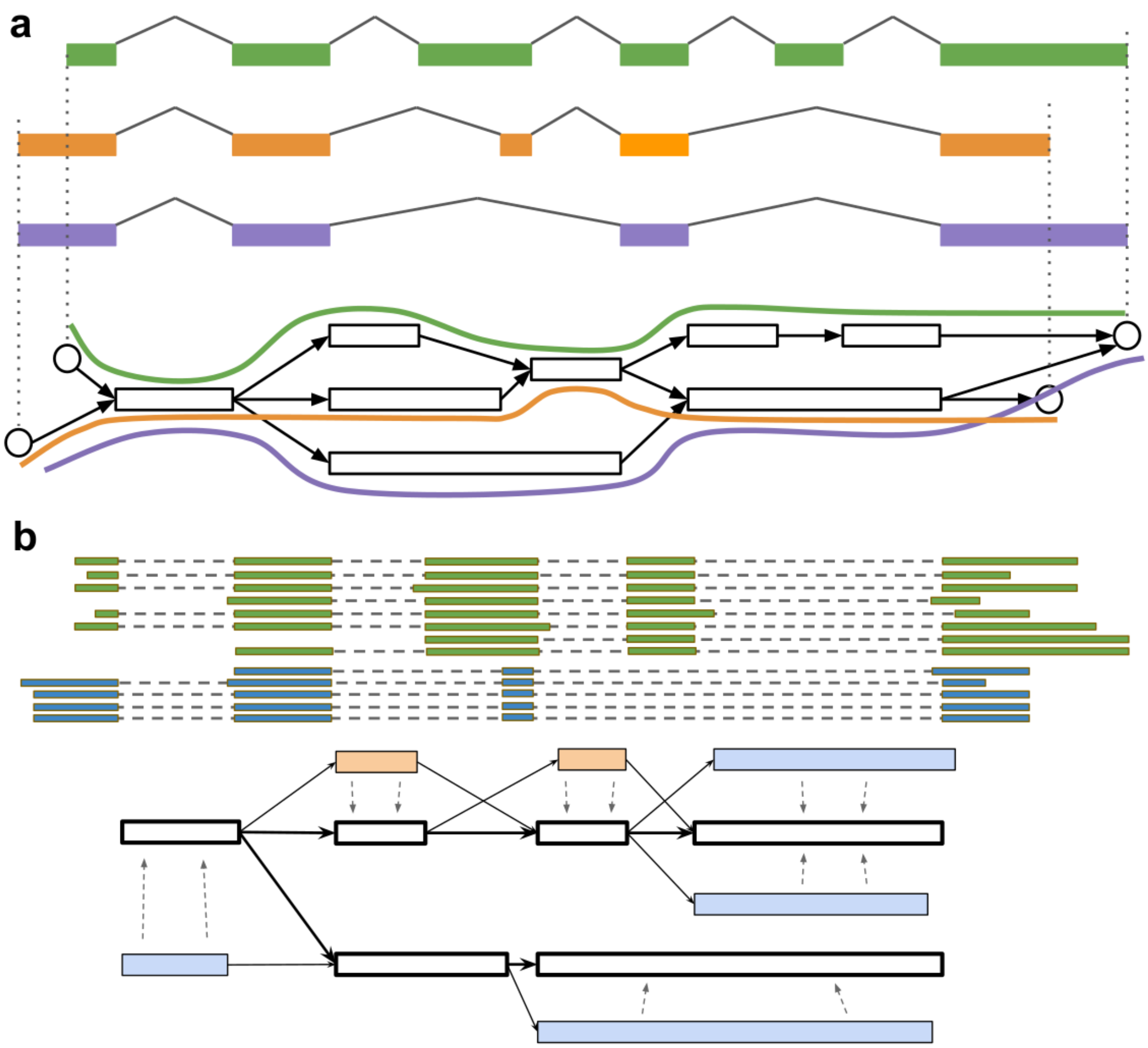

Supplementary Figure 6. Intron graph construction. a. An example of an intron graph constructed for a known gene having 3 isoforms (green, orange and purple). Each splice junction corresponds to an internal vertex in the graph (drawn as white rectangles), while graph edges connect adjacent splice junctions. Terminal vertices of the graph (drawn as circles) correspond to start and end positions of the transcripts. Each transcript can be represented as a path in the graph (colored lines). **b.** An example of an intron graph constructed from read alignments (without terminal vertices). White rectangles represent the correct splice junctions with a high read support, light blue and yellow vertices represent false splice junctions forming tips and bulges respectively. For each false splice junction that originates due to splice site misalignments, dashed arrows indicate the respective true splice junction that has both splice sites aligned correctly.

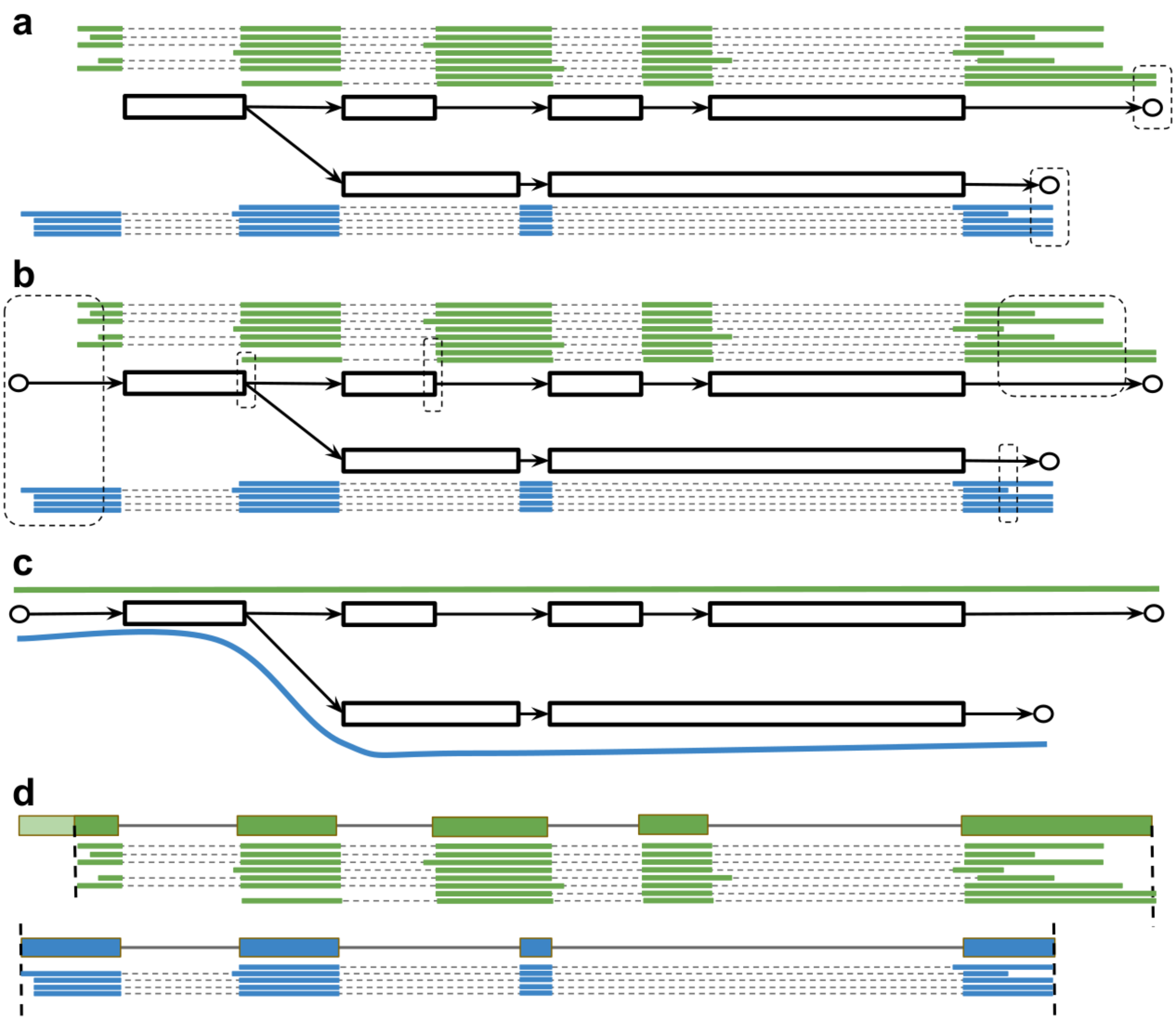

Supplementary Figure 7. Detection of terminal vertices and transcript reconstruction. In this example the gene is assumed to be on the forward strand. **a.** PolyA positions for each terminal splice junction are clustered and attached as terminal vertices in the simplified intron graph. **b.** Terminal non-polyA positions are not attached because the respective splice junctions have adjacent polyA vertices with larger genomic positions (right side). Leftmost splice junction is connected with a vertex representing the leftmost start position of all alignments (left side). Starting positions for other internal vertices are not selected since they are located either within or nearby 3’ of the preceding exons (in the middle). **c.** Paths representing full-length transcripts are constructed via read alignment traversal (color lines along the graph). **d.** Transcript ends are corrected using only consistent read alignments.

Supplementary Note 1: Investigating IsoQuant algorithms

The IsoQuant pipeline consists of several steps that involve multiple various algorithms. To provide more insights on the algorithm we evaluated the quality of novel transcripts generated by IsoQuant under different conditions.

We ran IsoQuant on the ONT R9.4 dataset and evaluated the quality of novel transcripts. We separately turned off various important steps of the algorithm, such as (i) splice site correction, (ii) transcript filtering based on relative read support, and (iii) intron clustering and graph simplification. As the Supplementary Figure 8 shows, of these 3 procedures the most dramatic effect on precision is caused by turning off clusterization and simplification procedures. Moreover, turning off all three simultaneously had a larger effect than the sum of the separate "turn-offs", suggesting that each of the three procedures can partially correct the mistakes of the other ones.

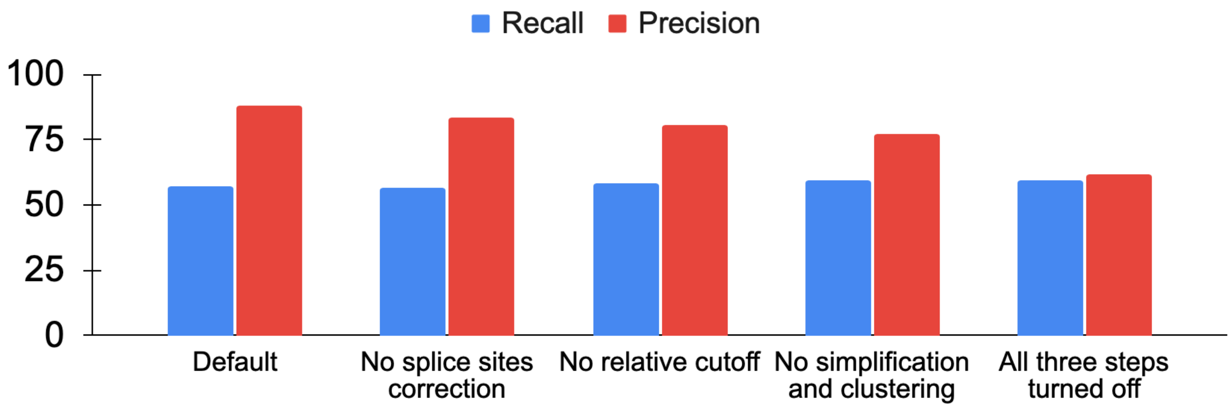

Supplementary Figure 8. Effect of turning off different IsoQuant procedures on overall performance. Recall (blue) and precision (red) are given for the novel transcripts generated by IsoQuant on ONT R9.4 simulated data with the reduced gene annotation.

	Delta, bp	0	3	6 (default)	12
All	Total	32,371	31,629	31,553	31,506
	Recall, %	**86**	85.1	85	84.9
	Precision, %	94.2	95.5	**95.6**	**95.6**
	F1-score	**0.90**	**0.90**	**0.90**	**0.90**
Novel	Total	4,031	3,875	3,848	3,820
	Recall, %	**63.4**	62.9	62.6	62.1
	Precision, %	83.4	86	**86.3**	86.2
	F1-score	0.72	**0.73**	**0.73**	0.72

Supplementary Table 10. IsoQuant performance on mouse ONT simulated data with different Delta values. The best values are indicated with bold.

IsoQuant has multiple internal parameters. One of the most important ones (Delta) defines allowed variability between splice junctions in reads. To understand the effect of this parameters we ran IsoQuant on mouse ONT simulated data with 4 different Delta values: 0 bp (no variation is allowed, each splice junction in the alignment is treated is the true one), 3 bp, 6 bp (default for ONT data) and 12 bp (Supplementary Table 10). Expectedly, for Delta=0 bp precision is lower compared to other values as any erroneously detected splice site may potentially be reported in the resulting annotation. At the same time, larger Delta values slightly decrease recall, as potential overcorrection of some splice sites may take place.

To allow a user to obtain annotations with higher recall in the cost of precision, we also implemented sensitive modes for both PacBio and ONT data. This will allow a user to change the behavior of the algorithm without tweaking multiple parameters. Supplementary Table 11 shows IsoQuant performance on mouse simulated data in default and sensitive modes.

	Metric	Default PacBio	Sensitive PacBio	Default ONT	Sensitive ONT
All	Total	33,549	33,857	31,553	32,010
	Recall, %	93.4	**98.5**	85	**85.7**
	Precision, %	**98.7**	94	**95.6**	95
	F1-score	**0.96**	**0.96**	**0.90**	**0.90**
Novel	Total	4,314	4,585	3,848	3,820
	Recall, %	76.8	**80.2**	62.6	**65.3**
	Precision, %	**94.4**	92.8	**86.3**	82.6
	F1-score	0.85	**0.86**	**0.73**	**0.73**

Supplementary Table 11. IsoQuant performance on mouse simulated data in default and sensitive mode. The best values are indicated with bold.

Supplementary Note 2: Varying the fraction of hidden transcripts

Since it is not clear how many novel isoforms may be present in the real RNA sample, we created several reduced human gene annotations with different fractions of excluded expressed transcripts: 10%, 15%, 20% and 25%. We launched each tool providing the same set of human simulated ONT reads, but different reduced annotations. Expectedly, for all tools both precision and recall of the entire output annotations gradually decrease as the proportion of hidden isoforms grows (Supplementary Figure 9a,b). Interestingly, the quality of novel transcripts alone hardly depends on this parameter. Moreover, some of the tools tend to predict transcripts even more accurately when a larger portion of reads represents unknown isoforms (Supplementary Figure 9c,d). It is worth noting that on this data IsoQuant also demonstrates the highest precision and recall on both novel and known transcripts independently of fraction of the annotation hidden.

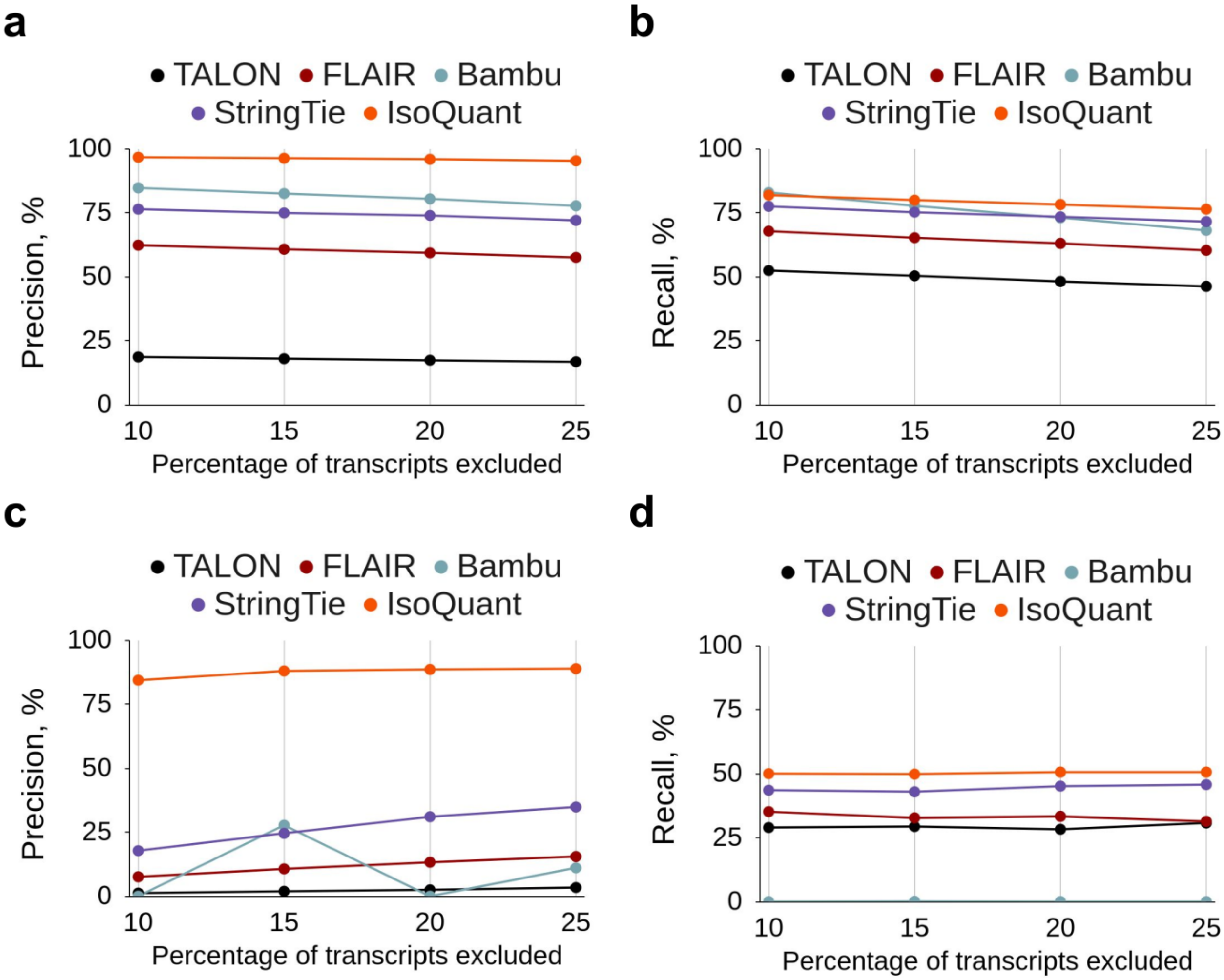

Supplementary Figure 9. Results for human ONT simulated data with the different reduced annotations. a. Precision of the entire output annotations for all 5 tools with respect to the percent of excluded transcripts. **b.** Same as (a), but for recall values. **c.** Precision of novel transcripts produced by all 5 tools with respect to the percent of excluded transcripts. **d.** Same as (c), but for recall values.

Supplementary Note 3: Varying simulation parameters

Simulation process takes an important role in the benchmarking process. Thus, understanding simulation parameters is essential for quality assessment. Beside transcript expression profiles that can be obtained using real data, simulation has two important parameters: sequencing error model and read truncation model. Here we first fixed the error model (ONT cDNA provided in NanoSim package) and varied read truncation probabilities by using (i) default NanoSim truncation, (ii) R9.4 truncation, (iii) R10.4 truncation and no truncation at all (see Supplementary Figure 2 for details). As Supplementary Table 12 shows, annotations obtained with the default NanoSim truncation are significantly worse as NanoSim dramatically over-truncates read sequences.

Further, we simulated data with different error models and fixed truncation probabilities. NanoSim package includes the default ONT cDNA error model, which was obtained using public human NA12878 ONT cDNA reads sequenced with r9.4 chemistry and has 15.9% overall error rate. We also trained NanoSim on our ONT R9.4 and R10.4 datasets and generated 2 models with error rates 11.5% and 2.8% respectively. As Supplementary Table 13 demonstrates, more accurate reads do increase precision of discovered transcript models. However, the effect looks rather marginal, as IsoQuant was initially designed to handle error-prone ONT data.

	Truncation	Default NanoSim truncation	R9.4 truncation	R10.4 truncation	No truncation
All	Total	24118	32243	32438	32373
	Recall, %	58.7	85.9	86	85.9
	Precision, %	86.4	94.6	94.1	94.1
	F1-score	0.70	0.90	0.90	0.90
Novel	Total	2556	3908	3999	3974
	Recall, %	21.9	63.2	63.1	62.7
	Precision, %	45.3	85.7	83.6	83.7
	F1-score	0.30	0.73	0.72	0.72

Supplementary Table 12. IsoQuant results on mouse ONT simulated data for fixed error model, but different truncation probabilities used during the simulation.

	Error model	R10.4	R9.4 (new basecalling)	R9.4 (NanoSim)
	Error rate	2.8%	11.5%	15.9%
All	Total	31553	32313	32438
	Recall, %	85	86.3	86
	Precision, %	95.6	94.7	94.1
	F1-score	0.90	0.90	0.90
Novel	Total	3848	4002	3999
	Recall, %	62.6	64	63.1
	Precision, %	86.3	84.8	83.6
	F1-score	0.73	0.73	0.72

Supplementary Table 13. IsoQuant results on mouse ONT simulated data for fixed truncation probabilities, but different error models.

Supplementary Note 4: Evaluating read-to-isoform assignment

Besides transcript prediction, IsoQuant is also capable of assigning aligned reads to the annotated transcripts. As the assignment is performed independently for each input sequence, we evaluated read-to-isoform assignment using PacBio and ONT reads simulated with the uniform expression profile (i.e., 10 reads per every annotated transcripts). We also benchmarked IsoQuant on reference transcript sequences and compared its performance with SQANTI3, which is designed specially for classifying transcript sequences. Both tools were provided with the comprehensive GENCODE annotation.

Precision and recall of the assigned reads were computed as follows. A read that is uniquely assigned to its correct isoform of origin was treated as a true positive call, while a unique assignment to a wrong isoform — as a false positive. Reads reported as ambiguous or inconsistent, as well as unassigned alignments were counted as false negatives. To compute precision and recall for the assignment algorithm alone (without taking into account mismapped reads), the same metrics were calculated only for the subset of reads that map to a correct gene of origin. The script for read-to-isoform assignment is located in the IsoQuant repository in misc/assess_assignment_quality.py.

Both IsoQuant and SQANTI3 classify reference transcripts with nearly perfect precision, while IsoQuant shows minor gain in recall. For simulated PacBio data, IsoQuant shows nearly the same performance compared to the reference transcripts, which can be explained by the relatively low error rate of PacBio CCS reads. For ONT data, however, assignment recall significantly drops down to 74.6%, most likely due to elevated error rate and higher percentage of truncated reads, which cannot be assigned to a known isoform unambiguously. Importantly, precision remains at a high level of 97.5% showing that IsoQuant can accurately assign long error-prone reads. Indeed, overall precision and recall values are slightly lower than the ones for the assignment algorithm alone, which indicates that in some cases incorrectly assigned or unassigned reads are caused by incorrect alignments (Supplementary Table 14).

Data	Reference transcripts		PacBio	ONT
Tool	SQANTI3	IsoQuant	IsoQuant	IsoQuant
Assignment precision	98.7	99.6	99.4	97.5
Assignment recall	91.3	97.6	96.9	74.6
Overall precision	97.5	98.2	98.9	96.3
Overall recall	89.1	95.6	92.3	70.3

Supplementary Table 14. Read-to-isoform assignment. Sequence assignment precision and recall for SQANTI3 (reference transcript only) and IsoQuant on mouse reference transcripts, PacBio CCS and ONT simulated data. Assignment precision and recall were calculated relative to the subset of sequences that map to their genes of origin. Overall statistics were computed using the entire set of input sequences.

Supplementary Note 5: Benchmarking transcript quantification

For quantification analysis we used simulated PacBio and ONT data with realistic expression profiles (35,684 expressed transcripts). The tools were provided with the respective comprehensive GENCODE annotation. As the novel isoform discovery algorithms are benchmarked in other experiments, here we estimated abundances only for the reference transcripts. StringTie was launched with the "-e" option, which turns off the detection of novel transcripts. IsoQuant was run with the default parameters and the reference transcript abundance table was used for further analysis ("*.transcript_tpm.tsv"). Reported TPM (transcript per million) values were compared with the true TPM values of the respective reference transcripts by computing: (i) Pearson correlation coefficient, (ii) the number of transcripts having TPM values within 10% (20%) range from the true reference values, (iii) the number transcripts that were falsely reported with non-zero expression, and (iv) the number of expressed transcripts with 0 TPM reported. When computing Pearson correlation coefficient both false reported and missed transcripts were included in the analysis. The script for computing these metrics can be found in misc/assess_quantification.py.

When using PacBio data IsoQuant reports accurate abundances with 93.8% of predicted TPM values falling within 20% range of their respective true TPM used during the simulation. StringTie, which was selected for this comparison as the tool with one the highest overall transcript precision, reports slightly less accurate expression levels: 77.7% of its TPM values fall within the 20% range and 15.7% of expressed transcripts are not reported at all. In contrast, IsoQuant missed only 2.5% of the expressed transcripts (Supplementary Table 15). For ONT data the reported expression levels appear to be less accurate as only 51.6% of TPM values reported by IsoQuant fall within the 20% range (and 41.4% for StringTie). Similarly to PacBio data, IsoQuant reports zero TPM for significantly fewer number of expressed transcripts (4.4% vs 20.7% for StringTie), but falsely reports more unexpressed transcripts (Supplementary Table 15).

	PacBio		ONT	
	StringTie	IsoQuant	StringTie	IsoQuant
# Transcripts with TPM > 0	30,246	34,919	30,951	40,091
Correlation coefficient	0.904	**0.944**	0.880	**0.893**
Within 10% range, %	67.4	**91.9**	26.6	**35.6**
Within 20% range, %	77.7	**93.8**	41.4	**51.6**
Missed, %	15.7	**2.5**	20.7	**4.4**
# Falsely detected	156	**144**	**2667**	5962

Supplementary Table 15. Quantification statistics for StringTie and IsoQuant on mouse simulated PacBio and ONT data when the full reference annotation is used. Percentage was computed with respect to true expressed transcripts. The best values are indicated with bold.

Supplementary Note 6: Testing IsoQuant with different spliced aligners

As the choice of the alignment software can dramatically affect the analysis quality, we evaluated IsoQuant with respect to (i) transcript model construction and (ii) read-to-isoform assignment with three different aligners: minimap2[1], deSALT[2], and uLTRA[3]. Since PacBio reads are accurate and are typically aligned correctly, for this experiment we used only mouse ONT R9.4 simulated data. The difference between the aligners in terms of read assignment is marginal. However, minimap2 allows to predict more precise transcript novel models. The vast majority of false positives in annotations obtained with uLTRA and deSALT alignments are mono-exonic transcripts that in IsoQuant are filtered using read mapping quality values, which seem to be less reliable for these two aligners. For multi-exonic transcripts however, both uLTRA and deSALT show false-positive rates comparable to minimap2. Importantly, minimap2 has significantly lower RAM requirements and running time, thus justifying the choice for the default aligner in the IsoQuant package.

	minimap2	deSALT	uLTRA
**Read-to-isoform assignment**			
Assignment recall	**74.6**	73.9	74.2
Assignment precision	**97.5**	**97.5**	97.1
Overall recall	70.3	**70.4**	70.1
Overall precision	**96.3**	95.8	95.7
**Transcript model construction**			
Recall of novel transcripts	**63.2**	60.3	60.9
Precision of novel transcripts	**85.7**	21.8	68.3
Overall recall	85.7	85.5	**86.9**
Overall precision	**94.9**	68.4	89.9
**Computational performance**			
Running time	**9h 29m**	17h 43m	80h 43m
Peak RAM, Gb	**24**	78	180

Supplementary Table 16. IsoQuant performance on mouse simulated ONT R9.4 data with different spliced aligners. Read-to-isoform analysis was assessed on data with uniform coverage. Transcript model construction and computational performance were evaluated on simulated data with the realistic coverage profile and the reduced gene annotation with 15% of isoforms hidden. The best values are indicated with bold.

Supplementary Note 7: Computational performance

While accuracy of delivered results often plays the most important role in the tool selection, computational performance and usability can be a significant criteria as well. Thus, we estimated running time and peak RAM consumption by all five tools used in this work on real human NA12878 ONT cDNA data. StringTie2, which is implemented in C++, dramatically outperforms all other tools by both parameters. Other tools show broadly comparable running time and memory consumption, with IsoQuant being slightly faster, but more greedy compared to FLAIR and Bambu (Supplementary Table 17). Importantly, minimap2, while being the fastest long-read spliced aligner among tested ones, takes more time than 4 out of 5 transcript construction tools assessed in this work. Since read mapping is a common step for every reference-based analysis, the difference in total running time of these 5 pipelines is less noticeable than for transcript discovery tools alone.

Tool	Wall clock time	CPU time	RAM peak, Gb
**Transcript construction**			
TALON	10h 27m	24h 56m	62
FLAIR	3h 04m	24h 10m	11
Bambu	3h 01m	8h 36m	20
StringTie	19m	57m	2
IsoQuant	2h 41m	23h 8m	24
**Alignment**			
minimap2	4h 46m	90h 58m	35

Supplementary Table 17. Computational performance. Running time and peak RAM for different tools on real human NA12878 ONT cDNA sequencing data. CPU time stands for total user and system processor time taken. All tools were launched on the same machine (120 cores, 1.5 TB RAM) in 20 threads.

Supplementary Note 8: Analysis of incorrectly reconstructed novel isoforms

To better understand what kind of errors are produced by each tool, we examined the output of gffcompare that provides the relationship between each reported isoform and the closest reference transcript. In simulated datasets (both PacBio and ONT), most errors in each tool were caused by using wrong splice junctions. Surprisingly, TALON outputs many isoforms that match with the reference transcripts on the opposite strand. Such behavior usually suggests mapping error, however, all tools were provided with the same BAM file, but this type of error was revealed predominantly in TALON.

Another major source of errors is isoform truncation, that is typical especially for TALON and FLAIR tools. Intron retention events were observed quite rarely, most of such events were produced by TALON using ONT data. IsoQuant errors were caused almost purely by using erroneous splice junctions. Bambu outputs a very low number of novel isoforms, so it is hard to characterize its typical errors.

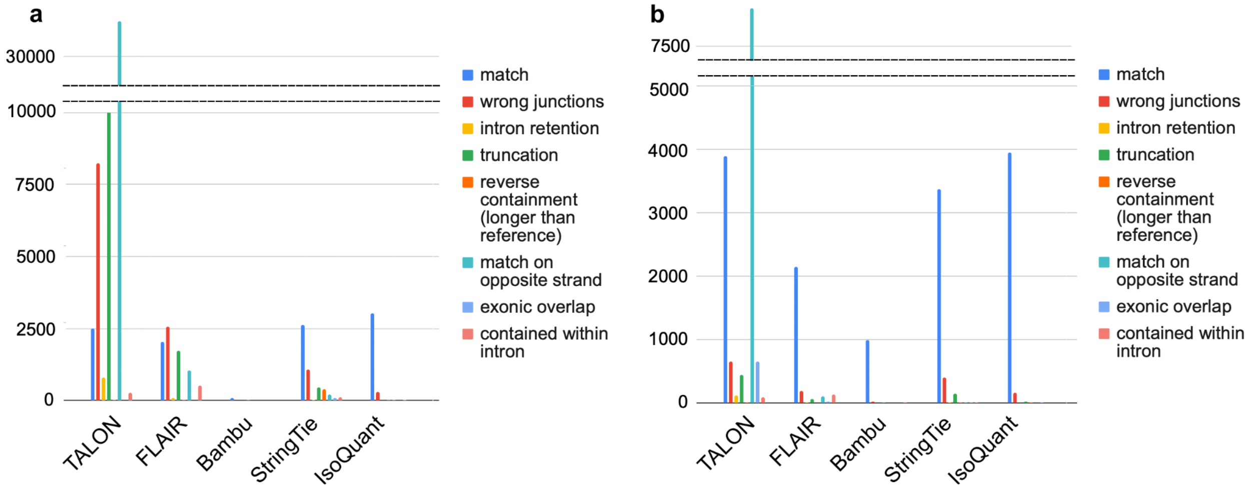

Supplementary Figure 10. Analysis of gffcompare output on ONT R10.4 (left) and PacBio (right) simulated datasets.

References

1. Li, H., 2018. Minimap2: pairwise alignment for nucleotide sequences. Bioinformatics, 34(18), pp.3094-3100.
2. Liu, B., Liu, Y., Li, J., Guo, H., Zang, T. and Wang, Y., 2019. deSALT: fast and accurate long transcriptomic read alignment with de Bruijn graph-based index. Genome biology, 20(1), pp.1-14.
3. Sahlin, K. and Mäkinen, V., 2021. Accurate spliced alignment of long RNA sequencing reads. Bioinformatics, 37(24), pp.4643-4651.
