# Supplementary material for: Accurate isoform discovery with IsoQuant using long reads
Source: Nat Biotechnol. 2023 Jan 2;41(7):915–8. doi: 10.1038/s41587-022-01565-y (PMC10344776; doi:10.1038/s41587-022-01565-y)
Supplement: Supplementary file 2 — Reporting Summary [file 41587_2022_1565_MOESM2_ESM.pdf]

Corresponding author(s): Hagen U Tilgner, Andrey Prjibelski

Last updated by author(s): Oct 6, 2022

Reporting Summary

Nature Research wishes to improve the reproducibility of the work that we publish. This form provides structure for consistency and transparency in reporting. For further information on Nature Research policies, see our Editorial Policies and the Editorial Policy Checklist.

Statistics

For all statistical analyses, confirm that the following items are present in the figure legend, table legend, main text, or Methods section.

n/a	Confirmed	
☒	☐	The exact sample size (*n*) for each experimental group/condition, given as a discrete number and unit of measurement
☒	☐	A statement on whether measurements were taken from distinct samples or whether the same sample was measured repeatedly
☒	☐	The statistical test(s) used AND whether they are one- or two-sided<br>*Only common tests should be described solely by name; describe more complex techniques in the Methods section.*
☒	☐	A description of all covariates tested
☒	☐	A description of any assumptions or corrections, such as tests of normality and adjustment for multiple comparisons
☒	☐	A full description of the statistical parameters including central tendency (e.g. means) or other basic estimates (e.g. regression coefficient) AND variation (e.g. standard deviation) or associated estimates of uncertainty (e.g. confidence intervals)
☒	☐	For null hypothesis testing, the test statistic (e.g. *F*, *t*, *r*) with confidence intervals, effect sizes, degrees of freedom and *P* value noted<br>*Give P values as exact values whenever suitable.*
☒	☐	For Bayesian analysis, information on the choice of priors and Markov chain Monte Carlo settings
☒	☐	For hierarchical and complex designs, identification of the appropriate level for tests and full reporting of outcomes
☒	☐	Estimates of effect sizes (e.g. Cohen's *d*, Pearson's *r*), indicating how they were calculated

Our web collection on statistics for biologists contains articles on many of the points above.

Software and code

Policy information about availability of computer code

Data collection	Simulated data was generated using modified NanoSim 3.0.0 and IsoSeqSim 2017-10-09 (both available at https://github.com/andrewprzh/lrgasp-simulation).
Data analysis	Open source software - Minimap2 (2.18), IsoQuant (3.0.1, https://github.com/ablab/IsoQuant), FLAIR (1.5), Bambu (2.0.0), StringTie (2.2.0), TALON (5.0), SQANTI (4.2), gffcompare (0.12.2), uLTRA (0.0.4.1), deSALT (1.5.6).

For manuscripts utilizing custom algorithms or software that are central to the research but not yet described in published literature, software must be made available to editors and reviewers. We strongly encourage code deposition in a community repository (e.g. GitHub). See the Nature Research guidelines for submitting code & software for further information.

Data

Policy information about availability of data

All manuscripts must include a data availability statement. This statement should provide the following information, where applicable:

- Accession codes, unique identifiers, or web links for publicly available datasets
- A list of figures that have associated raw data
- A description of any restrictions on data availability

Nanopore sequencing data obtained from the human NA12878 cell line is available at https://github.com/nanopore-wgs-consortium/NA12878/blob/master/RNA.md. PacBio human GM12878 data is available at ENCODE (https://www.encodeproject.org/search) under the accession numbers ENCFF450VAU and ENCFF694DIE. Sequencing data obtained from mouse brain samples is available at NCBI Gene Expression Omnibus (GEO; https://www.ncbi.nlm.nih.gov/geo/) under accession numbers GSE158450 and GSE178175. ONT SIRV data, simulated data and reduced gene annotations are published at https://zenodo.org/record/7121404.

Field-specific reporting

Please select the one below that is the best fit for your research. If you are not sure, read the appropriate sections before making your selection.

☒ Life sciences ☐ Behavioural & social sciences ☐ Ecological, evolutionary & environmental sciences

For a reference copy of the document with all sections, see nature.com/documents/nr-reporting-summary-flat.pdf

Life sciences study design

All studies must disclose on these points even when the disclosure is negative.

Sample size	This study involves no sample collection, therefore this field is not applicable.
Data exclusions	No data was excluded from the analysis.
Replication	No replicates were used in this study. Consistency and reproducibility of the developed tool was demonstrated on multiple different simulated and real datasets.
Randomization	This study involves no clinical or statistical experiments, randomization is not applicable.
Blinding	This study involves no clinical experiments or treatment, blinding is not applicable.

Reporting for specific materials, systems and methods

We require information from authors about some types of materials, experimental systems and methods used in many studies. Here, indicate whether each material, system or method listed is relevant to your study. If you are not sure if a list item applies to your research, read the appropriate section before selecting a response.

Materials & experimental systems

n/a	Involved in the study	
☒	☐	Antibodies
☐	☒	Eukaryotic cell lines
☒	☐	Palaeontology and archaeology
☒	☐	Animals and other organisms
☒	☐	Human research participants
☒	☐	Clinical data
☒	☐	Dual use research of concern

Methods

n/a	Involved in the study	
☒	☐	ChIP-seq
☒	☐	Flow cytometry
☒	☐	MRI-based neuroimaging

Eukaryotic cell lines

Policy information about cell lines

Cell line source(s)	HeLa cell line; source: the John Maciejowski lab (Sloan Kettering Institute), original commercial source: ATCC, catalog identifier: Cat#CRM-CCL-2.
Authentication	The cell line was not authenticated
Mycoplasma contamination	The cell line tested negative for mycoplasms
Commonly misidentified lines (See ICLAC register)	No commonly misidentified cell lines were used in this study.
